# Properties of Love Waves in Functional Graded Saturated Material

**DOI:** 10.3390/ma11112165

**Published:** 2018-11-02

**Authors:** Zhen Qu, Xiaoshan Cao, Xiaoqin Shen

**Affiliations:** 1 School of Science, Xi’an University of Technology, Xi’an 710054, China; quzhen@xaut.edu.cn; 2 School of Civil Engineering and Architecture, Xi’an University of Technology, Xi’an 710048, China; 3 State Key Laboratory of Transducer Technology, Chinese Academy of Sciences, Shanghai 200050, China

**Keywords:** functional graded saturated material, inhomogeneity, Love wave, dispersion, attenuation

## Abstract

In the present study, the propagation of Love waves is investigated in a layered structure with two different homogeneity saturated materials based on Biot’s theory. The upper layer is a transversely isotropic functional graded saturated layer, and the substrate is a saturated semi-space. The inhomogeneity of the functional graded layer is taken into account. Furthermore, the gradient coefficient is employed as the representation of the relation with the layer thickness and the material parameters, and the power series method is applied to solve the variable coefficients governing the equations. In this regard, the influence of the gradient coefficients of saturated material on the dispersion relations, and the attenuation of Love waves in this structure are explored, and the results of the present study can provide theoretical guidance for the non-destructive evaluation of functional graded saturated material.

## 1. Introduction

The research of the propagation characteristics of Love waves have been found in a wide range of engineering applications, such as seismic engineering, geotechnical engineering, and geophysics. Studies based on the elastic hypothesis have been sufficiently carried out. Since 1956, Biot [1,2,3] established the constitutive relation and the motion equation of saturated porous media. Based on Biot’s work, fruitful results have been yielded thereafter. Deresiewics et al. [4,5,6] derived the dispersion and attenuation equations of Love waves in the porous media. Wang, Tong, and Santos et al. [7,8,9] used the iteration method to solve the dispersion equation of porous materials. In addition, Konezak [10] and Ba et al. [11] gave a solution to the propagation of waves in porous layered half-space.

However, the research, which we have mentioned above, mainly focused on the homogeneous hypothesis of media. In the real situation, some saturated materials are always regarded as a layered and inhomogeneous medium, in which the material parameters vary continuously with the medium thickness. On this basis, how to explain the influence of homogeneity on wave propagation characteristics has become a crucial problem. In the recent years, some researchers use analytical methods to solve this problem. For example, Ke et al. [12] and Qian et al. [13] used the iterative method and Wentzel-Kramers-Brillouin (WKB) method, respectively, to deal with the inhomogeneity of materials, but have some limitations. In Ke’s work, the inhomogeneity of materials was described just as an exponential function, which we do not think is sufficient. The WKB method is too complicated for calculation. Cao et al. [14,15,16] used the power series method to solve Love wave and Rayleigh wave propagation problems in the FGM layered composite system.

In this study, the inhomogeneity of the saturated material and solid skeleton is supposed to be transversely isotropic. In addition, the assumption is made concerning the relationship between material thickness and material parameters, of which the latter vary continuously along the depth. Then, the dispersion relations and the attenuation of Love waves are investigated.

## 2. Statement of the Problem and Governing Equations

**Figure 1:**
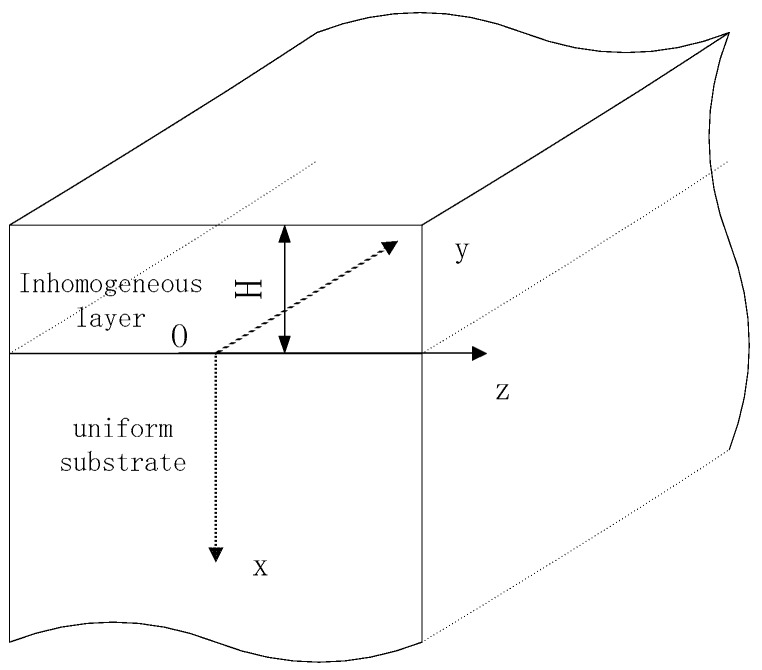
The functional graded saturated material layered structure.

The propagation of Love waves in a functional graded saturated media structure is shown in Figure 1. The upper layer is a transversely isotropic inhomogeneous saturated layer with the thickness of *H*. The surface of this layer is traction free, and the substrate is a homogeneous saturated half-space. Based on the Biot’s model of the homogeneous anisotropic saturated porous media, the soil skeleton is considered as a transversely isotropic medium. In terms of the Love waves propagation in the structure shown in Figure 1, the expressions of displacement are given as follows:

(1) $$\left\{ \begin{array}{l} u_{x}=u_{y}=0,\;u_{z}=u_{z}\left(x,y,t\right),\\ w_{x}=w_{y}=0,\;w_{z}=w_{z}\left(x,y,t\right). \end{array}\right.\;$$

Based on the motion equations, presented by Biot [3] in porous media, namely:

(2) $$\left\{ \begin{array}{l} \sigma_{ij,j}=\rho {\ddot{u}}_{i}+\rho_{f}{\ddot{w}}_{i},\\ -p_{f,j}=\rho_{f}{\ddot{u}}_{i}+m_{ii}{\ddot{w}}_{i}+r_{ii}{\dot{w}}_{i}, \end{array}\right.\;$$

where $p_{f}$ is the fluid pressure, and ρ is the density of saturated material, which can be expressed as $\rho =\left(1-\varphi \right)\rho_{s}+\varphi \rho_{f}$. $\rho_{s}$ is the density of solid skeleton, $\rho_{f}$ is the fluid density, and φ is the porosity of the solid. The $u_{i}$ in the equation is the component of the solid skeleton, and in terms of $w_{i}=-\varphi \left(u_{i}-U_{i}\right)$, $U_{i}$ is the displacement of fluid. The comma followed by the subscript i indicates the space differentiation with respect to the corresponding coordinate *x*, *y*, and *z*, the dot “•” represents time differentiation, and the repeated index is the means to summation related to that index. The parameter mii=Re[αi(ω)]ρf/φ and rii=η/Re[Ki(ω)] are Biot’s coefficients put forward by Biot. They are the functions of angular frequency ω and ω=ck. *C* and *k* are the velocity and numbers of the waves. Where η is the viscosity of the fluid, and $\alpha_{i}\left(\omega \right)$ and $K_{i}\left(\omega \right)$ are the dynamic tortuosity and permeability. Let $u_{x},\;u_{y},\;u_{z}$ and $w_{x},\;w_{y},\;w_{z}$ denote the displacement of the medium. The governing equations for the displacement of medium can be obtained.

Let ${\bar{u}}_{i},{\bar{w}}_{i}$ denote the displacement in the substrate layer. The expression of the governing equations for the Love waves propagating in the substrate layer (*x* > 0) are given as follows.

(3) $$\left\{ \begin{array}{l} {\bar{C}}_{44}\frac{\partial^{2}{\bar{u}}_{z}}{\partial x^{2}}+{\bar{C}}_{44}\frac{\partial^{2}{\bar{u}}_{z}}{\partial y^{2}}=\bar{\rho }\frac{\partial^{2}{\bar{u}}_{z}}{\partial t^{2}}+{\bar{\rho }}_{f}\frac{\partial^{2}{\bar{w}}_{z}}{\partial t^{2}},\\ {\bar{\rho }}_{f}\frac{\partial^{2}{\bar{u}}_{z}}{\partial t^{2}}+{\bar{m}}_{1}\frac{\partial^{2}{\bar{w}}_{z}}{\partial t^{2}}+{\bar{r}}_{1}\frac{\partial {\bar{w}}_{z}}{\partial t}=0, \end{array}\right.\;$$

where $C_{44}$ is the coefficient of material parameters. The “¯” symbol is used to denote the parameters in the substrate layer.

Similarly, we use ${\hat{u}}_{i},\;{\hat{w}}_{i}$ denote the displacement in the upper layer. The governing equations for the Love waves propagating in the upper layer (−*H* < *x* < 0) can be expressed as follows:

(4) $$\left\{ \begin{array}{l} {\hat{C}}_{44}\frac{\partial^{2}{\hat{u}}_{z}}{\partial x^{2}}+{\hat{C}}_{44}^{\prime }\frac{\partial {\hat{u}}_{z}}{\partial x}+{\hat{C}}_{44}\frac{\partial^{2}{\hat{u}}_{z}}{\partial y^{2}}=\hat{\rho }\frac{\partial^{2}{\hat{u}}_{z}}{\partial t^{2}}+{\hat{\rho }}_{f}\frac{\partial^{2}{\hat{w}}_{z}}{\partial t^{2}},\\ {\hat{\rho }}_{f}\frac{\partial^{2}{\hat{u}}_{z}}{\partial t^{2}}+{\hat{m}}_{1}\frac{\partial^{2}{\hat{w}}_{z}}{\partial t^{2}}+{\hat{r}}_{1}\frac{\partial {\hat{w}}_{z}}{\partial t}=0, \end{array}\right.\;$$

where the superscript “′” indicates the space differentiation with respect to the x− coordinate. The “^” symbol is used to denote the parameters in the upper layer, and these parameters are the functions of the x−axis, which needs to be emphasized.

The boundary condition of the present problem should be satisfied as follows: (a) the traction free boundary condition is ${\hat{\tau }}_{xz}\left(-H,y\right)=0$ at x=−H; (b) the stress and displacement are all continuous, ${\bar{\tau }}_{xz}\left(0,y\right)={\hat{\tau }}_{xz}\left(0,y\right)$, ${\bar{u}}_{z}\left(0,y\right)={\hat{u}}_{z}\left(0,y\right)$, ${\bar{w}}_{z}\left(0,y\right)={\hat{w}}_{z}\left(0,y\right)$; and (c) the attenuation conditions for Love waves are ${\bar{u}}_{z}\to 0$ at x→∞.

## 3. Solution of the Problem

In light of the present Love waves propagation problem we have discussed above, the solutions of the governing equations can be supposed as follows:

(5) $$\left\{ \begin{array}{l} u_{z}\left(x,y,t\right)=A_{z}\left(x\right)\exp\left[ik\left(y-ct\right)\right],\\ w_{z}\left(x,y,t\right)=W_{z}\left(x\right)\exp\left[ik\left(y-ct\right)\right], \end{array}\right.\;$$

where $i=\sqrt{-1}$, k=2π/λ is the wave number, and c is the phase velocity. $A_{z}\left(x\right)$ and $W_{z}\left(x\right)$ are the amplitudes of the displacement, which will be solved. Furthermore, the “¯” symbol and the “^” symbol are used to denote the substrate layer and the upper layer, respectively, so ${\bar{A}}_{z}\left(x\right)$ is the amplitudes of the displacement of the substrate layer, and ${\hat{A}}_{z}\left(x\right)$ refers to the amplitudes of the upper layer, respectively.

Firstly, in order to solve the problem in the substrate layer, we combine Equation (5) with Equation (3), and the governing equations can be modified as follows:

(6) $$\left\{ \begin{array}{l} {\bar{C}}_{44}\left({\bar{A}}_{z}^{\prime \prime }-k^{2}{\bar{A}}_{z}\right)=-\bar{\rho }c^{2}k^{2}{\bar{A}}_{z}-{\bar{\rho }}_{f}c^{2}k^{2}{\bar{W}}_{z},\\ {\bar{\rho }}_{f}c^{2}k^{2}{\bar{A}}_{z}+{\bar{m}}_{1}c^{2}k^{2}W_{z}+{\bar{r}}_{1}ick{\bar{W}}_{z}=0\;. \end{array}\right.\;$$

Then, the Love waves in the substrate layer can be expressed as follows:

(7) $$\left\{ \begin{array}{l} {\bar{u}}_{z}\left(x,y,t\right)=\left[C_{1}\exp\left(i\gamma x\right)+C_{2}\exp\left(-i\gamma x\right)\right]\exp\left[ik(y-ct)\right],\\ {\bar{w}}_{z}\left(x,y,t\right)=-\frac{{\bar{\rho }}_{f}ck}{{\bar{m}}_{1}ck+{\bar{r}}_{1}i}{\bar{u}}_{z}\left(x,y,t\right), \end{array}\right.\;$$

For the radiation condition of the Love waves, we must have **Im**(*γ*) > 0 and **Re**(*γ*) > 0. And, when integrating Equation (7) into the attenuation conditions, we can easily find that *C*_2_ = 0.

Secondly, the governing equations in the upper layer can be solved by combing Equation (5) with Equation (4), and the governing equation can be modified as follows:

(8) $$\left\{ \begin{array}{l} {\hat{C}}_{44}{\hat{A}}_{z}^{\prime \prime }+{\hat{C}}_{44}^{\prime }{\hat{A}}_{z}^{\prime }-{\hat{C}}_{44}k^{2}{\hat{A}}_{z}=-\hat{\rho }c^{2}k^{2}{\hat{A}}_{z}-{\hat{\rho }}_{f}c^{2}k^{2}{\hat{W}}_{z},\\ {\hat{\rho }}_{f}c^{2}k^{2}{\hat{A}}_{z}+{\hat{m}}_{1}ck{\hat{W}}_{z}+{\hat{r}}_{1}ick{\hat{W}}_{z}=0, \end{array}\right.\;$$

In order to solve the variable coefficient Equation (8), we assume the material parameters of the upper layer as the following functional form:

(9) $$\begin{array}{l} {\hat{C}}_{44}=\sum_{n=0}^{\infty }a_{n}^{1}{\left(\frac{x}{H}\right)}^{n},\hat{\rho }=\sum_{n=0}^{\infty }a_{n}^{2}{\left(\frac{x}{H}\right)}^{n},{\hat{\rho }}_{f}=\sum_{n=0}^{\infty }a_{n}^{3}{\left(\frac{x}{H}\right)}^{n},\\ {\hat{m}}_{1}=\sum_{n=0}^{\infty }a_{n}^{4}{\left(\frac{x}{H}\right)}^{n},{\hat{r}}_{1}=\sum_{n=0}^{\infty }a_{n}^{5}{\left(\frac{x}{H}\right)}^{n}, \end{array}$$

where the coefficients $a_{n}^{i}$ can be determined by the relations between the functions and their Taylor expansions. Then, the solutions of Equation (8) can be assumed to take the similar forms, as follows:

(10) $${\hat{A}}_{z}=\sum_{n=0}^{\infty }s_{n}{\left(\frac{x}{H}\right)}^{n},{\hat{W}}_{z}=\sum_{n=0}^{\infty }t_{n}{\left(\frac{x}{H}\right)}^{n}.\;$$

According to the integration of Equations (9) and (10) into Equation (8), the two recursive equations for $s_{n}$ and $t_{n}$ are presented as follows:

(11) $$\begin{array}{l} \sum_{i=0}^{n}\left(i+2\right)\left(i+1\right)a_{n-i}^{1}s_{i+2}+\sum_{i=0}^{n}\left(n-i+1\right)\left(i+1\right)a_{n-i+1}^{1}s_{i+1}-{\left(kH\right)}^{2}\sum_{i=0}^{n}a_{n-i}^{1}s_{i}\\ +c^{2}{\left(kH\right)}^{2}\sum_{i=0}^{n}a_{n-i}^{2}s_{i}+c^{2}{\left(kH\right)}^{2}\sum_{i=0}^{n}a_{n-i}^{4}t_{i}=0\;, \end{array}$$

(12) $$c^{2}k^{2}\sum_{i=0}^{n}a_{n-i}^{3}s_{i}+c^{2}k^{2}\sum_{i=0}^{n}a_{n-i}^{4}t_{i}+ick\sum_{i=0}^{n}a_{n-i}^{5}t_{i}=0\;.\;$$

We can calculate the coefficients of ${\left(x/H\right)}^{n}$, $s_{n}$, and $t_{n}$ with *n* from zero to infinity, using Equations (11) and (12). On this basis, a matrix is described to solve these coefficients.

(13) $$\left(s_{0j},s_{1j}\right)=I,\;$$

where *j* = 3~4 and **I** is a 2 × 2 unit matrix. The solution of Equation (8) can be rewritten as follows:

(14) $${\hat{A}}_{z}=\sum_{j=3}^{4}C_{j}\sum_{n=0}^{\infty }s_{nj}{\left(\frac{x}{H}\right)}^{n},{\hat{W}}_{z}=\sum_{n=0}^{\infty }t_{n}{\left(\frac{x}{H}\right)}^{n}.\;$$

According to the discussion we have made above, the solution of Equation (4) can be described as follows:

(15) $$\left\{ \begin{array}{l} {\hat{u}}_{z}\left(x,y,t\right)=\left[\sum_{j=3}^{4}C_{j}\sum_{n=0}^{\infty }S_{nj}{\left(\frac{x}{H}\right)}^{n}\right]\exp\left[ik\left(y-ct\right)\right],\\ {\hat{w}}_{z}\left(x,y,t\right)=\left[\sum_{n=0}^{\infty }t_{n}{\left(\frac{x}{H}\right)}^{n}\right]\exp\left[ik\left(y-ct\right)\right], \end{array}\right.\;$$

Then, we apply Equations (15) and (7) to the boundary condition of the present problem, and there are a set of homogeneous linear algebraic equations of unknown coefficients *C_i_*,*i* = 1, 3, 4 obtained. According to the condition for the existence of a non-trivial solution, the determinant of the coefficients matrix Q must be vanished.

(16) |Q|=0. 

## 4. Numerical Results and Discussion

The numerical examples will be given to illustrate the propagation characters of Love waves in the functional graded saturated layer, which are lying on a homogeneous saturated soil half-space. First and foremost, some important hypotheses must be introduced. In light of our problem, we used the following expression [7] to calculate the *α*(*ω*) and *K*(*ω*).

(17) $$\eta {\left[\omega K_{i}\left(\omega \right)\right]}^{-1}=i\varphi^{-1}\rho_{f}\alpha_{i}\left(\omega \right)=i\varphi^{-1}\rho_{f}\alpha_{i}\left(\infty \right)\left[1+\frac{4if_{ci}}{3f}\times {\left(1-\frac{3i}{8}\frac{f}{f_{ci}}\right)}^{1/2}\right],\;$$

where f=ω/2π is the wave frequency. $f_{ci}=\omega_{ci}/2\pi =3\eta \varphi {\left[8\pi K_{i}\left(0\right)\alpha_{i}\left(\infty \right)\rho_{f}\right]}^{-1}$ is a critical frequency, which was reported by Sharma in 1991 [17]. At the functional graded layer, the material parameters are functions of layer thickness, and these functions can be assumed as follows:

(18) g=1−exp(px/H) , 

where the parameter *p* is the gradient coefficient, which refers to the level of layer inhomogeneity. On this basis, the parameter function of soil thickness can be described as follows:

(19) $${\hat{C}}_{44}={\bar{C}}_{44}\cdot g,\;$$

and the other parameters in the upper layer have the similar forms.

In the present paper, the influence of the gradient coefficient on the Love waves dispersion relations and attenuation will be discussed. In detail, from the governing Equation (3), Equation (4), and dispersion relations Equation (16), the wave number *k* in our problem is a complex *k* = *k*_1 +_ *k*_2_. Then, the dispersion relation curves will be drawn as the relation between the phase velocities *c* and *k*_1_ in convenient, and we designate *δ* = *k*_2_*/k*_1_ as the attenuation coefficient to evaluate the Love wave attenuation in our problem. In order to solve the complex dispersion equation, we used the method called the minimum modulus value approximation, in order to approximate the suitable solution. The theme of this method is described below. We assume the material parameter of the homogeneous saturated media as follows: ${\bar{C}}_{44}=4\;\mathrm{Gpa}$, $\bar{\varphi }=0.2$, ${\bar{K}}_{1}(0)=1$, ${\bar{\alpha }}_{1}(\infty )=1$, $\eta =10^{-3}\;\mathrm{pa}\cdot s$, ${\bar{\rho }}_{s}=30\;\mathrm{kN}/m^{3}$, ${\bar{\rho }}_{l}=10\;\mathrm{kN}/m^{3}$, ${\bar{\rho }}_{g}=1.2\;\mathrm{kN}/m^{3}$.

### 4.1. Influence of the Gradient Coefficient on Love Wave Dispersion

In order to describe the influence of the gradient coefficient on the Love wave dispersion, it is necessary to give a solution to the complex Equation (16). First of all, according to the research conducted by Sharma [17] and Wang [7], the Love wave speed has a range in the porous medium that is determined by a critical frequency, $f_{ci}$. In this paper, we also chose them as the method to calculate the range of the Love wave speed for specific gradient coefficients. Secondly, based on the range of speed, we employed the minimum modulus value approximation method to obtain the suitable solution of Equation (16). The theme of this method should be given as follows: (a) for a given speed range of the specific gradient coefficient *p* and *n*th modes of Love wave, we choose four values of (*k*_1_*H*,*k*_2_*H*) from *k*_1_*H* = 0, and made them as a square; (b) calculate the determinant of Equation (16); (c) choose the values (*k*_1_*H*,*k*_2_*H*), which have the minimum value of determinant and use (*k*_1_*H*,*k*_2_*H*) as an angular point to make the new square, which has a half-length of the side of the previous square; (d) repeat the step (c) until the value of determinant reaches zero; and (e) give an increment of *k*_1_*H*, and repeat the whole procedures. Then, we can draw a dispersion curve of the *n*th mode of the Love wave. At the same time, the attenuation coefficient $\log[k_{2}/k_{1}]$ can also be calculated in the given gradient coefficient *p* and *n*th modes of wave.

**Figure 2:**
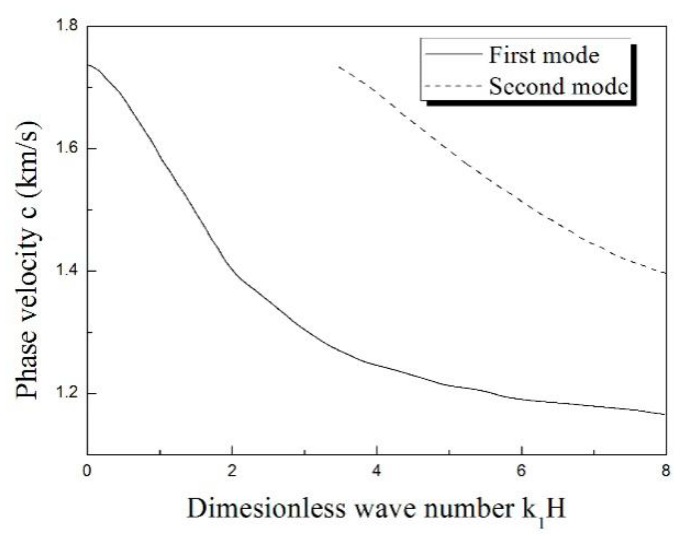
The dispersion curve of the Love wave in the inhomogeneous unsaturated layer lies on homogeneous saturated half-space.

**Figure 3:**
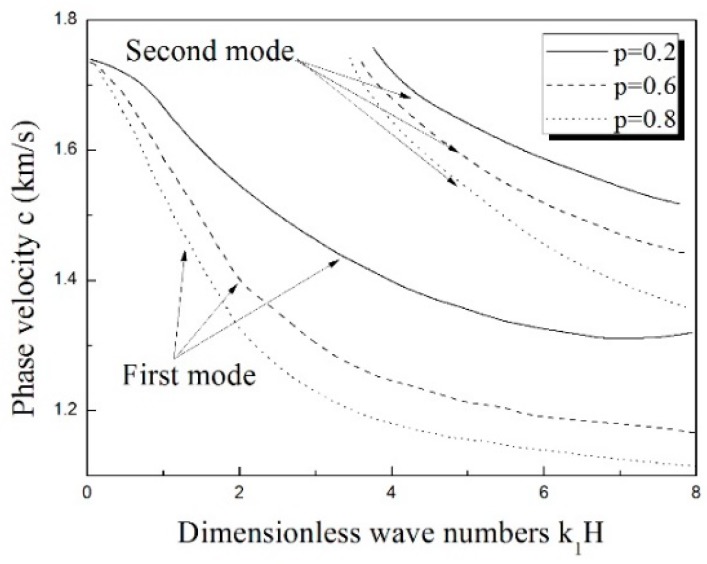
The comparison of the dispersion relation of the Love wave with the different gradient parameter *p*.

**Figure 4:**
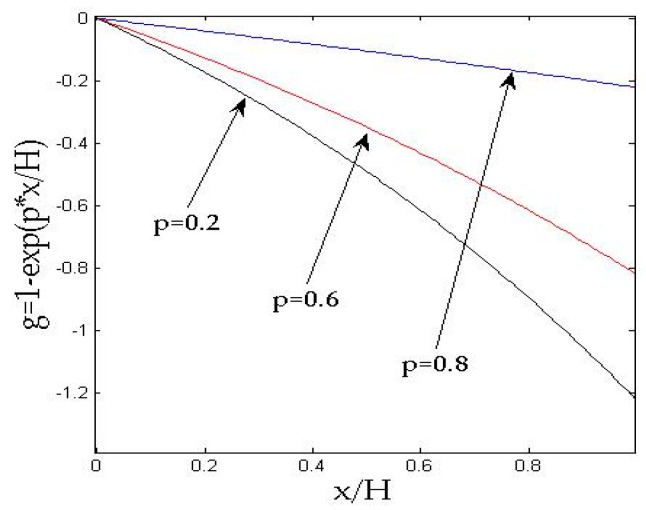
The material parameter distributions.

Figure 2 presents the Love wave dispersion curve of 1st and 2nd modes with the gradient *p* = 0.6. The comparison of the different gradient coefficients (*p* = 0.2, 0.6, 0.8) is shown in Figure 3. The results in Figure 3 suggest that the gradient coefficient *p* gives a conspicuous impact of Love wave dispersion. And Figure 4 shows the material parameter distributions. With the increase of the gradient coefficient *p*, the phase velocity of the Love wave decreases obviously, and the influence of the gradient coefficient on the first mode is more intense than that on the second mode. For the first mode of the Love wave, with the increase of dimensionless wave numbers *k*_1_*H*, the influence of gradient coefficients on phase velocity gradually increases. In terms of the second mode of the Love wave, the influence is smoother than that on the first mode.

### 4.2. Influence of the Gradient Coefficient on Love Wave Attenuation

**Figure 5:**
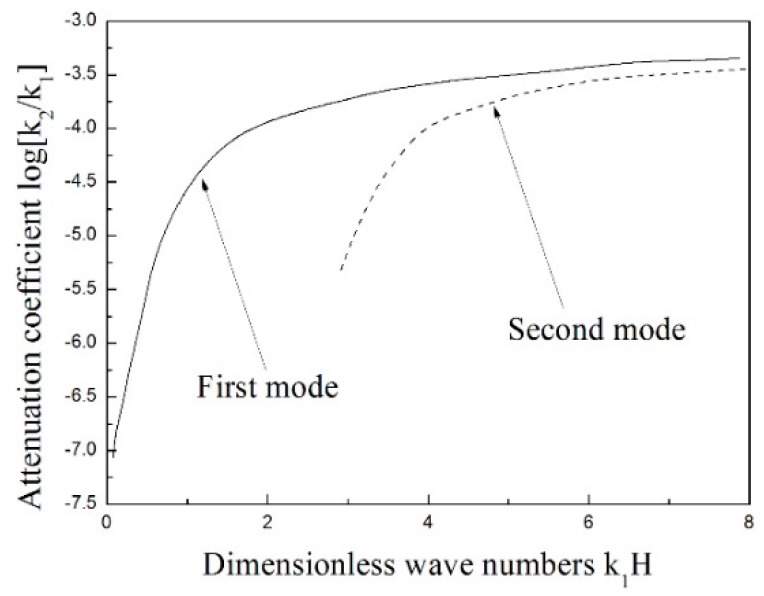
The attenuation curve of the Love wave in the inhomogeneous saturated layer lies on homogeneous saturated half-space.

**Figure 6:**
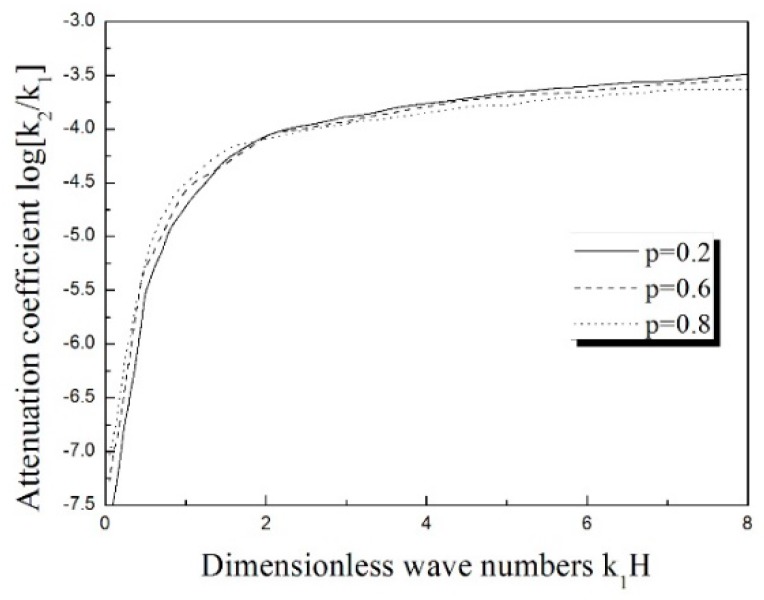
The comparison of the attenuation of the Love wave with the different gradient parameter *p*.

The attenuation of the Love wave is shown in Figure 5. The solid line denotes the first mode attenuation, and the second mode is expressed by the dashed line. As the two modes indicate, the attenuation rapidly increases at first, and then becomes smoother with the increase of dimensionless wave numbers, *k*_1_*H*. The investigating results of the influence of the gradient coefficient on the attenuation of the Love wave are plotted in Figure 6. In the current study, the discussion mainly focuses on the situation of the Love wave attenuation in the first mode. The solid line refers to the situation of *p* = 0.2, the dashed line describes the *p* = 0.6, and the case of *p* = 0.8 is plotted as the dotted line. It is easily seen that the change of gradient coefficient almost exerts no effect on the Love wave attenuation, and the influence of material inhomogeneity on the attenuation of wave is very little. In this regard, great interest is entailed in the comparison with the rapid influence of inhomogeneity on the dispersion of the Love wave. 

## 5. Conclusions

In this paper, based on the Biot’s saturated porous medium theory, the influence of inhomogeneity has been theoretically analyzed on the propagation character of the Love wave in a transversely isotropic inhomogeneous saturated layer lying on a saturated half-space. The governing equations of the problem have been solved by the power series method, and the minimum modulus value approximation method is employed to discuss the dispersion equation of the Love wave. The gradient coefficient *p* has been introduced to describe the inhomogeneity of the saturated media, and we obtained the dispersion and attenuation curve of the Love wave with different gradient coefficients. It is important to note that the gradient coefficient has a great influence on the dispersion of the Love wave, but the effect of the gradient coefficient on the attenuation is less significant.

## References

[1] Biot M.A. (1955). Theory of Elasticity and Consolidation for a porous Anisotropic Solid. J. Appl. Phys..

[2] Biot M.A. (1956). General solutions of the equation of elasticity and consolidation for a porous material. J. Appl. Phys..

[3] Biot M.A. (1962). Generalized theory of acoustic propagation in porous dissipative media. J. Acoust. Soc. Am..

[4] Deresiewisz H. (1961). The effect of boundaries on wave propagation in a liquid-filled porous solid—II. Love waves in a porous layer. Bull. Seismol. Soc. Am..

[5] Deresiewisz H. (1964). The effect of boundaries on wave propagation in a liquid-filled porous solid—VI. Love waves in double surface layer. Bull. Seismol. Soc. Am..

[6] Deresiewisz H. (1974). The effect of boundaries on wave propagation in a liquid-filled porous solid—XI. Waves in a plate. Bull. Seismol. Soc. Am..

[7] Wang Y.S., Zhang Z.M. (1998). Propagation of Love waves in transversely isotropic fluid-saturated porous layered half-space. J. Acoust. Soc. Am..

[8] Tong L.H., Liu Y.S., Geng D.X., Lai S.K. (2007). Nonlinear wave propagation in porous materials based on the Biot theory. J. Acoust. Soci. Am..

[9] Santos J.E., Corbero J., Lovera O.M. (2016). A model for wave propagation in a porous medium saturated by a two-phase fluid. J. Acoust. Soc. Am..

[10] Konezak Z. (1989). The propagation of Love waves in a fluid-saturated porous anisotropic layer. Acta Mech..

[11] Ba Z.N., Liang J.W., Lee V.W. (2016). Wave propagation of buried spherical SH-, P1-, P2- and SV-waves in a layerd poroelastic half-space. Soil Dyn. Earthq. Eng..

[12] Ke L.L., Wang Y.S., Zhang Z.M. (2006). Love waves in an inhomogeneous fluid saturated porous layered half-space with linearly varying properties. Soil Dyn. Earthq. Eng..

[13] Qian Z.H., Jin F., Kishimoto K., Lu T.J. (2009). Propagation behavior of Love waves in a functionally graded half-space with initial stress. Int. J. Solids Struct..

[14] Cao X.S., Jin F., Jeon I., Lu T.J. (2009). Propagation of Love waves in a functionally graded piezoelectric material layered composite system. Int. J. Solids Struct..

[15] Cao X.S., Jin F., Jeon I. (2011). Calculation of propagation properties of lamb waves in a functionally graded material plate by power series technique. NDT&E Int..

[16] Cao X.S., Jin F., Kishimoto K. (2012). Transverse Shear surface wave in functionally graded material infinite half space. Philos. Mag. Lett..

[17] Sharma M.D., Gogna M.L. (1991). Wave propagation in anisotropic liquid saturated porous solids. J. Acoust. Soc. Am..

